# Simultaneous Occurrence of Erythema Nodosum in Monozygotic Twin Sisters

**DOI:** 10.1155/2012/109427

**Published:** 2012-06-05

**Authors:** Farhanag Babamahmoodi, Abdolreza Babamahmoodi, Hamidreza Barani, Leila Delavarian

**Affiliations:** ^1^Department of Infectious Diseases, Mazandaran University of Medical Sciences, Sari, Iran; ^2^Health Management Research Center, Baqiyatallah University of Medical Sciences, Tehran, Iran

## Abstract

Erythema nodosum (EN) is the most frequent clinicopathologic variant of panniculitis with painful red or violaceous nodules on the anterior surfaces of the legs. The condition is a cutaneous reaction that might be associated with a wide variety of disorders or might be caused by medications that produce painful nodules on the shins, and less commonly on the thighs and forearms. In this paper, we describe, for the first time in the world, erythema nodosum as the simultaneous presenting complaint of monozygotic twin sisters after streptococcal pharyngitis. This paper might support the effect of heredity in the occurrence of erythema nodosum.

## 1. Introduction

Erythema nodosum (EN) is clinically presented as an acute, ill-defined, nonulcerative, erythematous. and painful eruption, which is usually confined to the extensor aspects of lower extremities. Females comprise the majority of the patients. Erythema nodosum is probably the most common type of panniculitis and a delayed hypersensitivity reaction to a variety of antigens. The condition might be associated with a wide variety of etiologic factors, such as medications, infections, systemic inflammatory diseases, malignancies, sarcoidosis, rheumatologic disorders, inflammatory bowel diseases, pregnancy, and so on [1]. In 60% of EN cases, no cause is found [2].

Typically, EN is manifested by the sudden onset of symmetrical, tender, erythematous, warm nodules, and raised plaques, usually located on the shins, ankles, and knees and the lesions are often distributed bilaterally [2]. Diagnosis is usually based on a compatible clinical presentation. In patients with atypical features, deep skin biopsy is necessary for confirmation. The typical pathologic presentation is septal panniculitis, with infiltration of neutrophils, lymphocytes, and even multinucleated giant cells in the periseptal areas of fat lobules. Vascular deposition of immunoglobulin complexes and complement has been hypothesized as the etiologic factors involved in septal panniculitis, but evidence of this pathophysiological process remains elusive [1]. Treatment of EN should be directed to the underlying associated condition if identified. In most patients, EN is a self-limited disease and usually nodules of EN regress spontaneously within a few weeks, requiring only symptomatic relief using nonsteroidal anti-inflammatory drugs, potassium iodide, cool wet compresses, elevation, and bed rest. Systemic corticosteroids are rarely indicated in EN, and an underlying infection should be ruled out before they are administered [1–3].

## 2. Case Report

**Figure 1:**
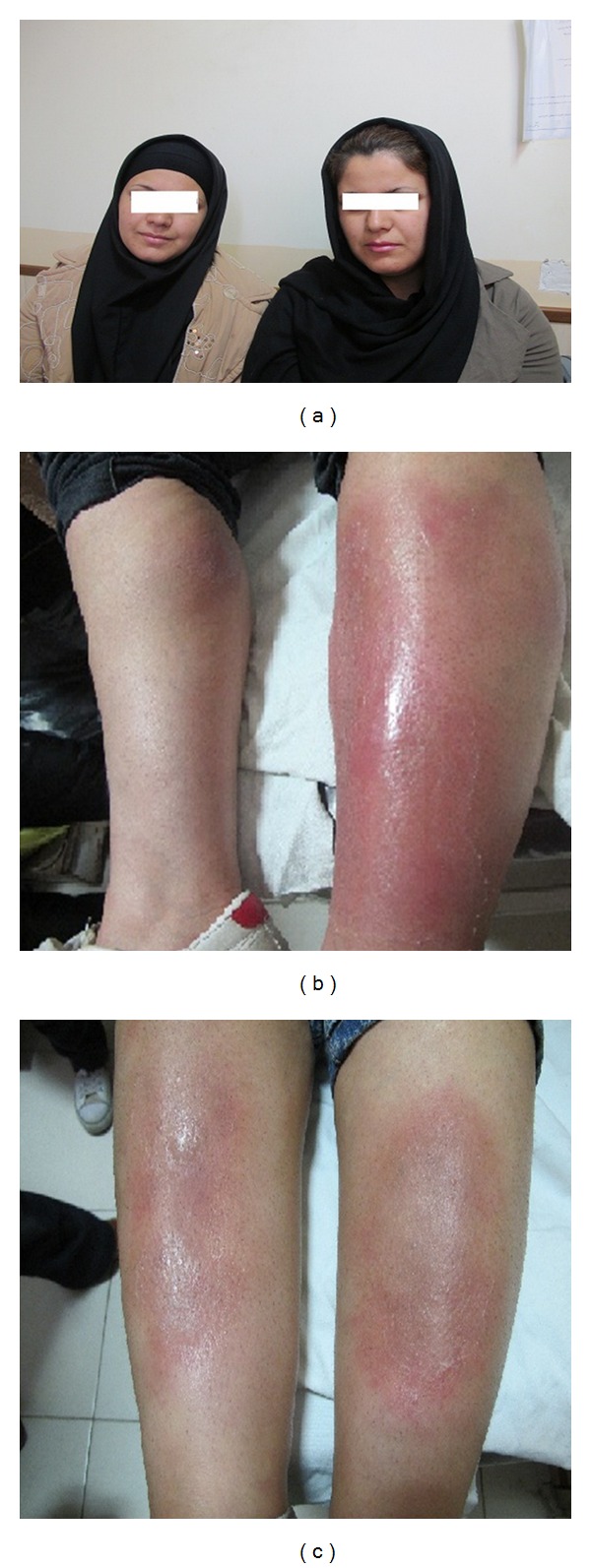
Skin lesions: case 1 on the right side and case 2 on the left side.

In this case report, for the first time in the world, we describe erythema nodosum as the simultaneous presenting complaint of monozygotic twin sisters after streptococcal pharyngitis (Figure 1(a)).

It should be noted that both patients provided their informed consent.

### 2.1. Case One

A married 22-year-old lady presented with a chief complaint of tenderness, heat, and pain in the anterior parts of both legs of one-week duration; no pitting edema was present. The signs had gradually developed on the lower one-third of the right leg in an area 3–5 cm in diameter and on the left leg in an area 4-5 cm in diameter. In the physical examination, the patient was ill without fever and erythema, and nodular lesions were seen (Figure 1(b)).

### 2.2. Case Two

A married 22-year-old lady presented with a chief complaint of pain and tenderness on the anterior part of her right leg, which gradually increased in intensity; the same problems appeared on the left leg, too. Physical examination revealed 0.5 × 0.5 cm nodules with 3–50 cm erythematous margins. She was ill but afebrile (Figure 1(c)).

Both the patients reported sore throat, fever, chills, and general symptoms of a cold two weeks previously and reported taking oral contraceptives. Physical examination did not yield any abnormal findings. After 8 weeks, the conditions completely resolved. During the sickness period the patients received a single dose of penicillin 1200000 U intramuscularly and nonsteroidal anti-inflammatory drugs. After 8 weeks the conditions completely resolved.

## 3. Laboratory Findings

Table 1 presents the laboratory data of the two cases.

## 4. Discussion

The eruptive phase of erythema nodosum starts with flulike symptoms of fever and generalized aches and pains. Arthralgia might occur and appear before or during the eruptive phase. Most lesions in infection-induced erythema nodosum ameliorate within 7 weeks, but active disease may last up to 18 weeks. In contrast, 30% of idiopathic erythema nodosum cases may last more than 6 months. Febrile illness with dermatologic findings includes abrupt onset of illness with initial fever, followed by a painful rash within 1-2 days [4]. When erythema nodosum is diagnosed, it is important to find out the underlying conditions. These include a detailed history, including drug and past medical history, a careful physical examination, laboratory investigation, and a chest X-ray.

EN is classified as idiopathic or secondary to other diseases [5]. Some causes of erythema nodosum and its differential diagnosis are listed in Table 1. There are various etiologic factors for erythema nodosum based on the disease pattern of the region. Table 2 presents some retrospective studies that show this variation based on geographical differences [3, 6–9]. 

The relationship between a previous episode of upper respiratory tract infection by group A beta-hemolytic streptococci and erythema nodosum is fully known, particularly in children and young adults. Usually, the cutaneous lesions appear 2-3 weeks after throat infection, and they are associated with an increase in antistreptolysin O (ASO) titer. An intradermal positive test to streptococcal antigens is often found in patients with erythema nodosum secondary to streptococcal infections, although when the cutaneous nodules develop, the cultures of ordinary throat swabs usually do not detect microorganisms [1, 10].

Drugs are frequently implicated as the cause of erythema nodosum. Sulfonamides, bromides, and oral contraceptives have long been established as the most common medications contributing to EN. In those patients who develop erythema nodosum when an antibiotic is taken for an infectious disease it is difficult to determine whether the cutaneous reaction is due to the antibiotic or due to the infectious agent [1, 11].

In publications and data bases, there is no report about simultaneous occurrence of erythema nodosum in monozygotic twins or in a family. Almost none of the studies reveal anything about genetics and heredity in the etiology of EN. Labunski et al. reported that patients with erythema nodosum associated with sarcoidosis produce an uncommon tumor necrosis factor (TNF-II). These patients show a nucleotide exchange, (G-A) at position 308 in the human TNF gene promoter, whereas patients with erythema nodosum without underlying sarcoidosis display a similar allele frequency compared with controls [12]. These results support the notion that erythema nodosum, in association with sarcoidosis, might be pathogenetically linked to altered TNF-*α* production due to a genetic promoter polymorphism [1, 3, 5]. In contrast, other authors have found that the proinflammatory cytokine pattern exhibits increased interleukin-6 serum concentrations both in infectious and noninfectious disease-related erythema nodosum, whereas a minor involvement of TNF is found in these patients [1, 11].

Our paper might help researchers elucidate the pathogenesis of EN. In addition, this paper might have diagnostic value in identifying patients with specific conditions associated with EN.

## Figures and Tables

**Table 1:** Laboratory data of the two cases.

Tests	Case one	Case two	Normal value
Throat culture	Strep B hemolytic group A	Strep B hemolytic group A	Negative
Cold agglutination	Negative	Negative	lower than 1 : 32
White blood cell	10100	7400	4.3–10.8 × 10^3^/mm^3^
Neutrophil	72%	70%	45–74%
Red blood cell	3.58	4.22	3.5–5 × 10^6^/mcL
Hemoglobin	9.6	11.4	12.1–15.3 g/dL
Platelets	315000	334000	150–450 × 10^3^/mcL
Erythrocyte sedimentation rate	75	39	30 mm/hr
C-reactive protein	2+	1+	no CRP detectable
ASO	400	200	less than 160 Todd units per milliliter
PPD	Negative	Negative	Negative
Chest X-ray	Normal	Normal	Normal

**Table 2:** Retrospective studies that show variation according to geographical differences.

Author	Year	Number of cases	Country	Idiopathic	Infection
Carlos et al. [6]	2000	106 patients (82 females)	Spain	35%	34%
Psychos et al.[3]	2000	110 females	Greece	35%	17.3%
Tay [7]	2000	75 patients (65 females)	Singapore	60%	29%
Puavilai et al.[8]	1995	100 patients (88 females)	Thailand	72%	18%
